# Supplementary material for: Two chemically distinct root lignin barriers control solute and water balance
Source: Nat Commun. 2021 Apr 19;12:2320. doi: 10.1038/s41467-021-22550-0 (PMC8055973; doi:10.1038/s41467-021-22550-0)
Supplement: Supplementary file 1 — Supplementary information [file 41467_2021_22550_MOESM1_ESM.pdf]

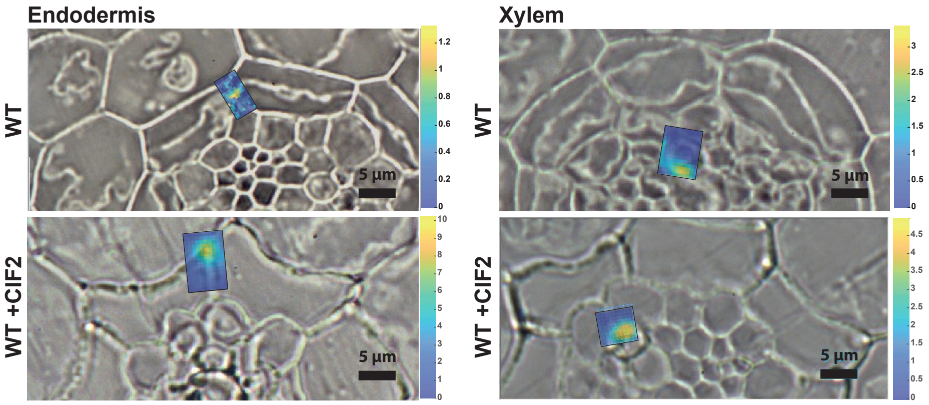

Supplementary Fig. 1. Activation of the Schengen-pathway triggers the deposition of a distinct "stress" lignin in the endodermis.
Examples of small Raman maps for endodermal cells of root cross-sections in WT(Ø) and WT(+CIF2) and for xylem of WT(Ø) and WT(+CIF2) used for determining the lignin spectra using Multivariate Curve Resolution (MCR) presented in Fig. 2c, d. The colour code represents the intensity of the lignin factor presented in Fig. 2c, d. Similar small Raman maps were obtained from independent plants for CS lignin of WT (n = 8 plants), cell-corner lignin of WT treated with CIF2 (+CIF2; n = 5 plants), for xylem lignin of WT (n = 2 plants) and xylem lignin of WT treated with CIF2 (n = 2 plants).

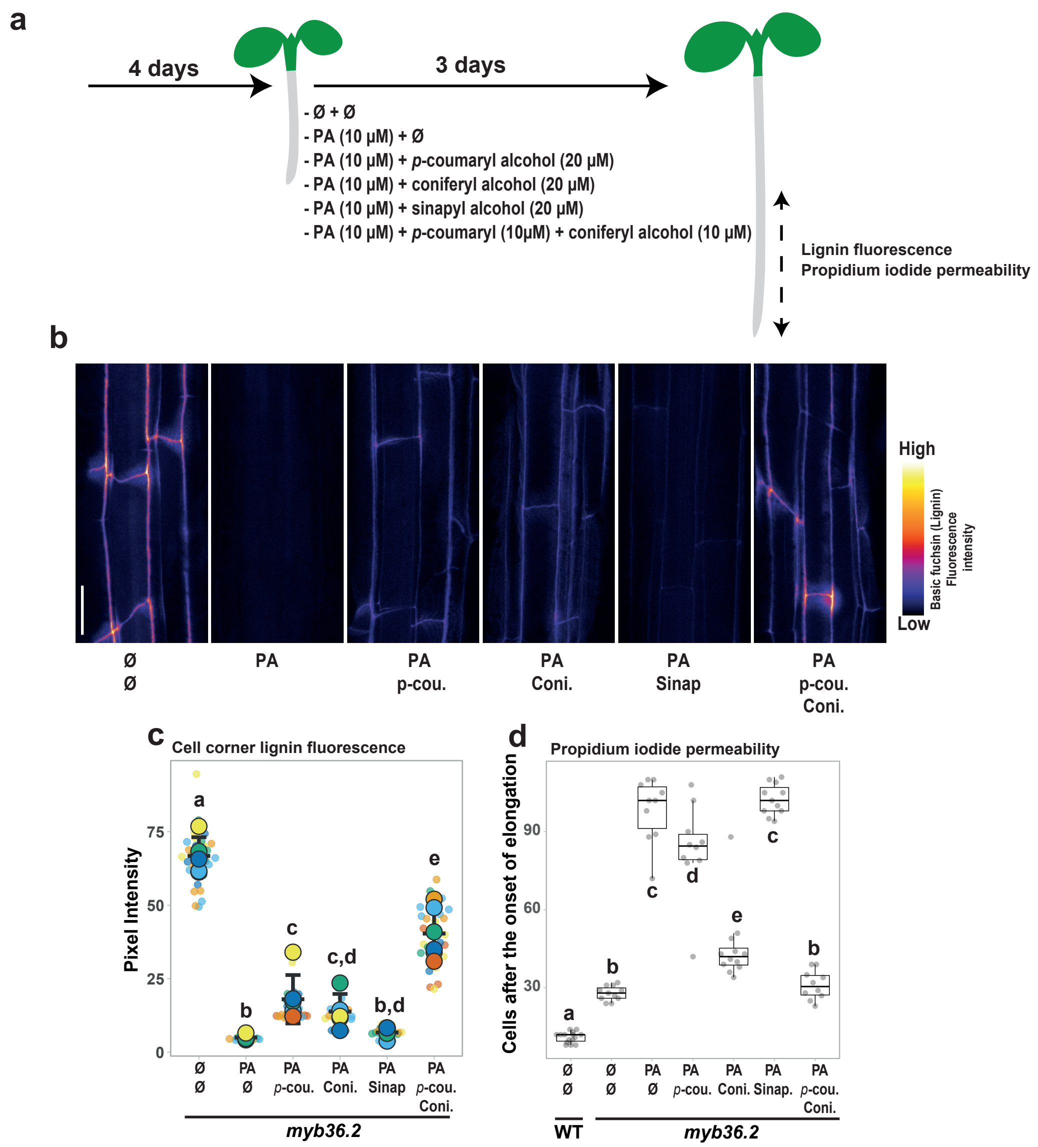

Supplementary Fig. 2. The lignin compositional changes induced by the Schengen pathway contributes to the sealing of the apoplast.

a Scheme of experimental design. The *myb36-2* mutant was germinated for 4 days on control condition and then transferred for three more days on a control media (Ø + Ø), on a media containing 10 µM piperonylic acid only (PA + Ø), or supplemented with 20 µM *p*-coumaryl alcohol (PA + *p*-cou.), with 20 µM coniferyl alcohol (PA + Coni.), with 20 µM sinapyl alcohol (PA + Sinap.) and with the combination of 10 µM *p*-coumaryl alcohol and 10 µM coniferyl alcohol (PA + p-cou + Coni.). **b** Maximum intensity projection of the top endodermal cells at ~20 cells after the onset of elongation in roots stained with Basic fuchsin (lignin). Scale bar = 25 µm. Representative pictures are shown. The experiment was repeated two times independently with similar results. **c** Fluorescence intensity of lignin stained using Basic fuchsin in the cortex-facing cell wall corner of endodermal cells at ~20 cells after the onset of elongation in *myb36-2*. Individual data points (small dots) represent the pixel intensity for one endodermal cell. Measurements of pixel intensity were performed in 3 to 6 individual cells per plants and measurements were performed in 5 plants for (Ø + Ø), 4 plants for (PA + Ø), 6 plants for (PA + *p*-cou.), 5 plants for (PA + coni.), 5 plants for (PA + Sinap.) and 6 plants for (PA + *p*-cou + Coni.). Large dots represent the mean of pixel intensity measured in each plant. Black lines represent the mean and standard deviation for each treatment. Letters show significantly different treatments determined by an ANOVA and Tukey's test as post hoc analyses ($p < 0.01$). **d** Boxplot showing the number of cells from the onset of elongation permeable to propidium iodide in wild-type (WT) and *myb36.2* in control condition (Ø + Ø) and after pharmacological treatments described in (a) for *myb36.2*. Measurements were performed in 15 plants for WT (Ø + Ø) WT, 11 plants for *myb36.2* (Ø + Ø), 10 plants for *myb36.2* (PA + Ø), 10 plants for *myb36.2* (PA + *p*-cou.), 12 plants for *myb36.2* (PA + p-coni.), 11 plants for *myb36.2* (PA + Sinap.) and 10 plants for *myb36.2* (PA + *p*-cou + Coni.). Center lines show the medians; box limits indicate the 25th and 75th percentiles.Different letters represent significant differences between treatments using a two-sided Mann-Whitney test ($p<0.05$).

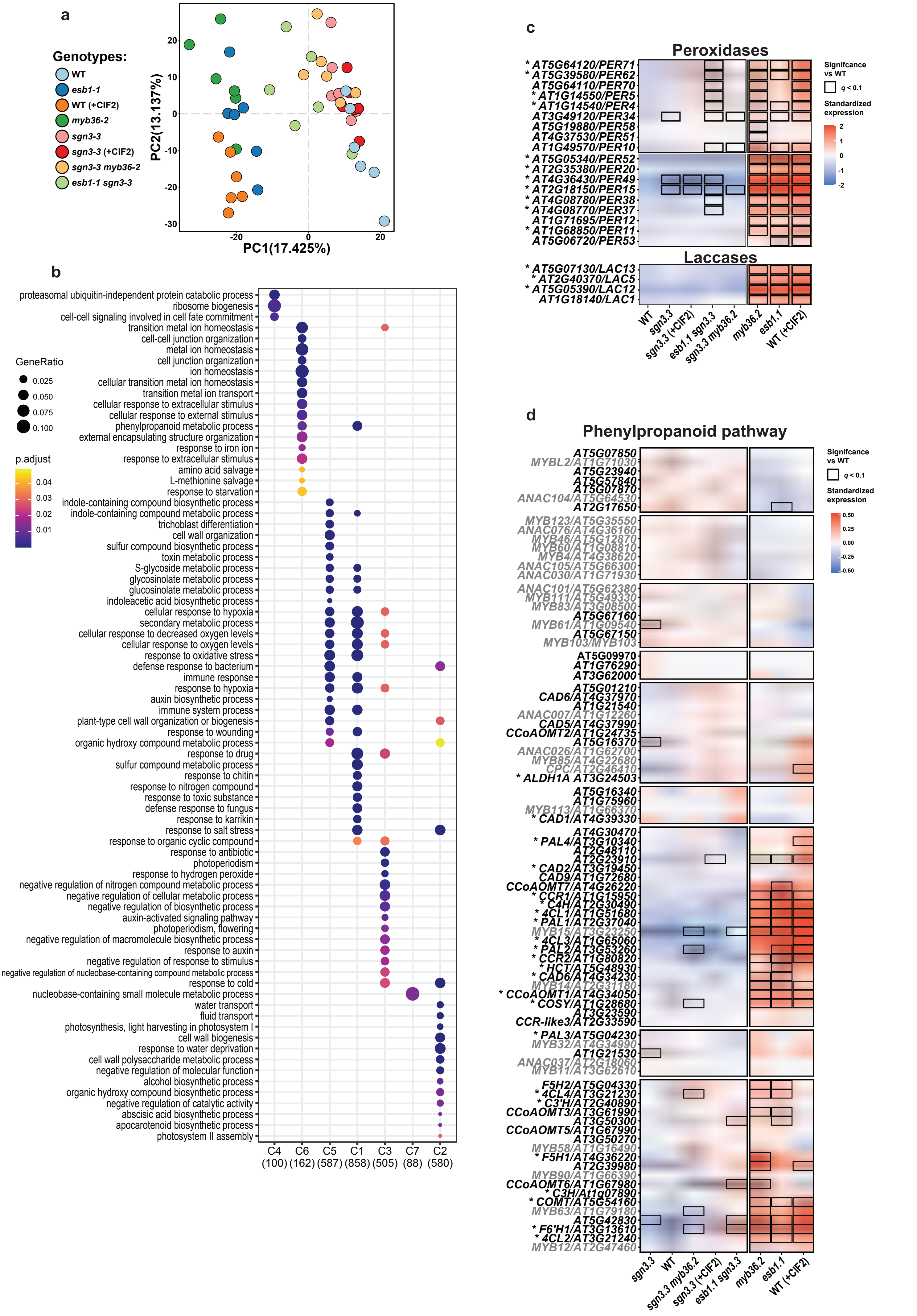

a
Genotypes:
WT
esb1-1
WT (+ClF2)
myb36-2
sgn3-3
sgn3-3 (+ClF2)
sgn3-3 myb36-2
esb1-1 sgn3-3
PC2(13.137%)
PC1(17.425%)
b
GeneRatio
0.025
0.050
0.075
0.100
p.adjust
0.04
0.03
0.02
0.01
proteasomal ubiquitin-independent protein catabolic process
ribosome biogenesis
cell-cell signaling involved in cell fate commitment
transition metal ion homeostasis
cell-cell junction organization
metal ion homeostasis
cell junction organization
ion homeostasis
cellular transition metal ion homeostasis
transition metal ion transport
cellular response to extracellular stimulus
cellular response to external stimulus
phenylpropanoid metabolic process
external encapsulating structure organization
response to iron ion
response to extracellular stimulus
amino acid salvage
L-methionine salvage
response to starvation
indole-containing compound biosynthetic process
indole-containing compound metabolic process
trichoblast differentiation
cell wall organization
sulfur compound biosynthetic process
toxin metabolic process
S-glycoside metabolic process
glycosinolate metabolic process
glucosinolate metabolic process
indoleacetic acid biosynthetic process
cellular response to hypoxia
secondary metabolic process
cellular response to decreased oxygen levels
cellular response to oxygen levels
response to oxidative stress
defense response to bacterium
immune response
response to hypoxia
auxin biosynthetic process
immune system process
plant-type cell wall organization or biogenesis
response to wounding
organic hydroxy compound metabolic process
response to drug
sulfur compound metabolic process
response to chitin
response to nitrogen compound
response to toxic substance
defense response to fungus
response to karrikin
response to salt stress
response to organic cyclic compound
response to antibiotic
photoperiodism
response to hydrogen peroxide
negative regulation of nitrogen compound metabolic process
negative regulation of cellular metabolic process
negative regulation of biosynthetic process
auxin-activated signaling pathway
photoperiodism, flowering
negative regulation of macromolecule biosynthetic process
response to auxin
negative regulation of response to stimulus
negative regulation of nucleobase-containing compound metabolic process
response to cold
nucleobase-containing small molecule metabolic process
water transport
fluid transport
photosynthesis, light harvesting in photosystem I
cell wall biogenesis
response to water deprivation
cell wall polysaccharide metabolic process
negative regulation of molecular function
alcohol biosynthetic process
organic hydroxy compound biosynthetic process
negative regulation of catalytic activity
abscisic acid biosynthetic process
apocarotenoid biosynthetic process
photosystem II assembly
C4 (100)
C6 (162)
C5 (587)
C1 (858)
C3 (505)
C7 (88)
C2 (580)
c
Peroxidases
* AT5G64120/PER71
* AT5G39580/PER62
AT5G64110/PER70
* AT1G14550/PER5
* AT1G14540/PER4
AT3G49120/PER34
AT5G19880/PER58
AT4G37530/PER51
AT1G49570/PER10
* AT5G05340/PER52
* AT2G35380/PER20
* AT4G36430/PER49
* AT2G18150/PER15
* AT4G08780/PER38
* AT4G08770/PER37
AT1G71695/PER12
* AT1G68850/PER11
AT5G06720/PER53
Laccases
* AT5G07130/LAC13
* AT2G40370/LAC5
* AT5G05390/LAC12
AT1G18140/LAC1
WT
sgn3.3
sgn3.3 (+ClF2)
esb1.1 sgn3.3
sgn3.3 myb36.2
myb36.2
esb1.1
WT (+ClF2)
Signfcance vs WT
q < 0.1
Standardized expression
2
1
0
-1
-2
d
Phenylpropanoid pathway
AT5G07850
MYBL2/AT1G71030
AT5G23940
AT5G57840
AT5G07870
ANAC104/AT5G64530
AT2G17650
MYB123/AT5G35550
ANAC076/AT4G36160
MYB46/AT5G12870
MYB60/AT1G08810
MYB4/AT4G38620
ANAC105/AT5G66300
ANAC030/AT1G71930
ANAC101/AT5G62380
MYB111/AT5G49330
MYB83/AT3G08500
AT5G67160
MYB61/AT1G09540
AT5G67150
MYB103/MYB103
AT5G09970
AT1G76290
AT3G62000
AT5G01210
CAD6/AT4G37970
AT1G21540
ANAC007/AT1G12260
CAD5/AT4G37990
CCoAOMT2/AT1G24735
AT5G16370
ANAC026/AT1G62700
MYB85/AT4G22680
CPC/AT2G46410
* ALDH1A AT3G24503
AT5G16340
AT1G75960
MYB113/AT1G66370
* CAD1/AT4G39330
AT4G30470
* PAL4/AT3G10340
AT2G48110
AT2G23910
* CAD2/AT3G19450
CAD9/AT1G72680
CCoAOMT7/AT4G26220
* CCR1/AT1G15950
* C4H/AT2G30490
* 4CL1/AT1G51680
* PAL1/AT2G37040
MYB15/AT3G23250
* 4CL3/AT1G65060
* PAL2/AT3G53260
* CCR2/AT1G80820
* HCT/AT5G48930
* CAD6/AT4G34230
MYB14/AT2G31180
* CCoAOMT1/AT4G34050
* COSY/AT1G28680
AT3G23590
CCR-like3/AT2G33590
* PAL3/AT5G04230
MYB32/AT4G34990
AT1G21530
ANAC037/AT2G18060
MYB11/AT3G62610
F5H2/AT5G04330
* 4CL4/AT3G21230
* C3'H/AT2G40890
CCoAOMT3/AT3G61990
AT3G50300
CCoAOMT5/AT1G67990
AT3G50270
MYB58/AT1G16490
* F5H1/AT4G36220
AT2G39980
MYB90/AT1G66390
CCoAOMT6/AT1G67980
* C3H/At1g07890
* COMT/AT5G54160
MYB63/AT1G79180
AT5G42830
* F6'H1/AT3G13610
* 4CL2/AT3G21240
MYB12/AT2G47460
sgn3.3
WT
sgn3.3 myb36.2
sgn3.3 (+ClF2)
esb1.1 sgn3.3
myb36.2
esb1.1
WT (+ClF2)
Signfcance vs WT
q < 0.1
Standardized expression
0.50
0.25
0.00
-0.25
-0.50

Supplementary Fig. 3. Gene expression profiling in response to the activation of the Schengen-pathway.

a Principal component analysis (PCA) of the differentially expressed genes identified in root tips of wild-type (WT), *sgn3-3*, *esb1-1*, *myb36-2*, *esb1-1 sgn3-3*, *sgn3-3 myb36-2* plants. Treatment with 100 nM CIF2 was applied as indicated (+CIF2) for WT and *sgn3-3* plants (n = 6 biological replicates from two independents experiments). **b** Gene ontology enrichment in the different gene clusters from Fig. 3a. The colour of each point represents the p-value adjusted using the Benjamin-Hochberg procedure, and the size of each point denotes the percentage of total differential expressed genes in the given gene ontology term (Gene Ratio). **c** Heatmap of gene expression for peroxidases and laccases that are upregulated by the constitutive activation of the Schengen pathway as defined by the Cluster 1 (C1) of the Fig. 3a. Asterisks indicate genes previously identified as upregulated in response to CIF2[1]. **d** Heatmap of gene expression of genes related to the phenylpropanoid pathway (black)[2] and their transcriptional regulators (grey)[3,4]. Genes names are given according to[5] for genes related to the phenylpropanoid pathway. Asterisks indicate demonstrated function in lignin biosynthesis with an activity demonstrated *in vitro* or *in vivo* for *PAL1-4*[6], *C4H*[7], *4CL1-4*[8,9], *CCR1* and *2*[10,11], *CAD1*, 2 and *6*[12,13], *C3'H*[14], *C3H*[15], *COMT* and *CCoAOMT1*[16], *HCT*[17], *CSE*[18], *ALDH1A*[19], *F6'H1*[20], *COSY*[21] and *F5H1*[22].

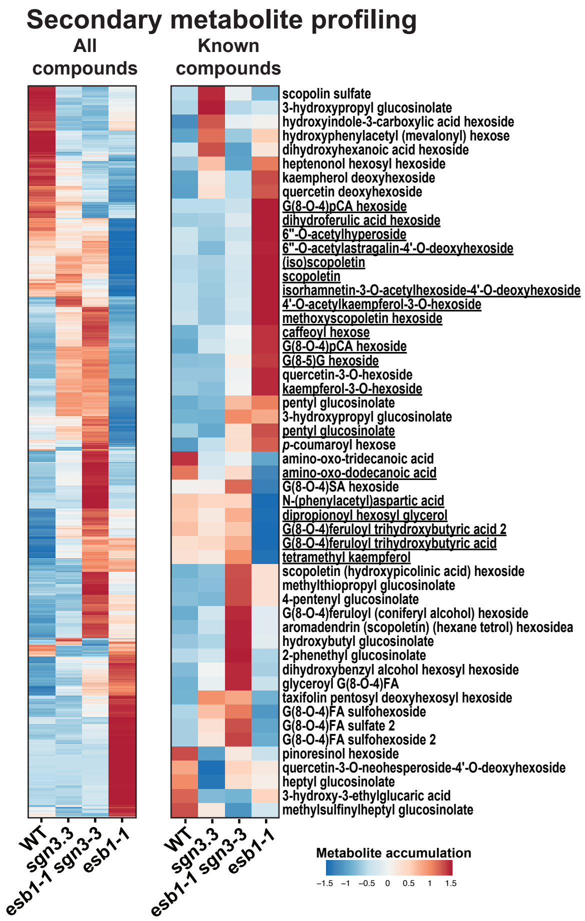

Supplementary Fig. 4. Metabolite profiling in response to the activation of the Schengen-pathway.

Heatmaps of metabolite profiling determined using Ultra High Performance Liquid Chromatography (UHPLC) in 5 mm roots tips of wild-type (WT), *sgn3-3*, *esb1-1 sgn3-3* and *esb1-1*. The heatmaps show all the compounds (2497, left) and characterised compounds (52, right) that are differentially accumulated (q-value < 0.01, left; q-value < 0.1, right n = 8 biological replicates from two independent experiments). Underlined names are for compounds that are only differentially accumulated (q-value < 0.1) in *esb1-1* and not changed in *sgn3-3* and *esb1-1 sgn3-3* in comparison with WT. Data for the known compounds are presented in Supplementary Data 3.

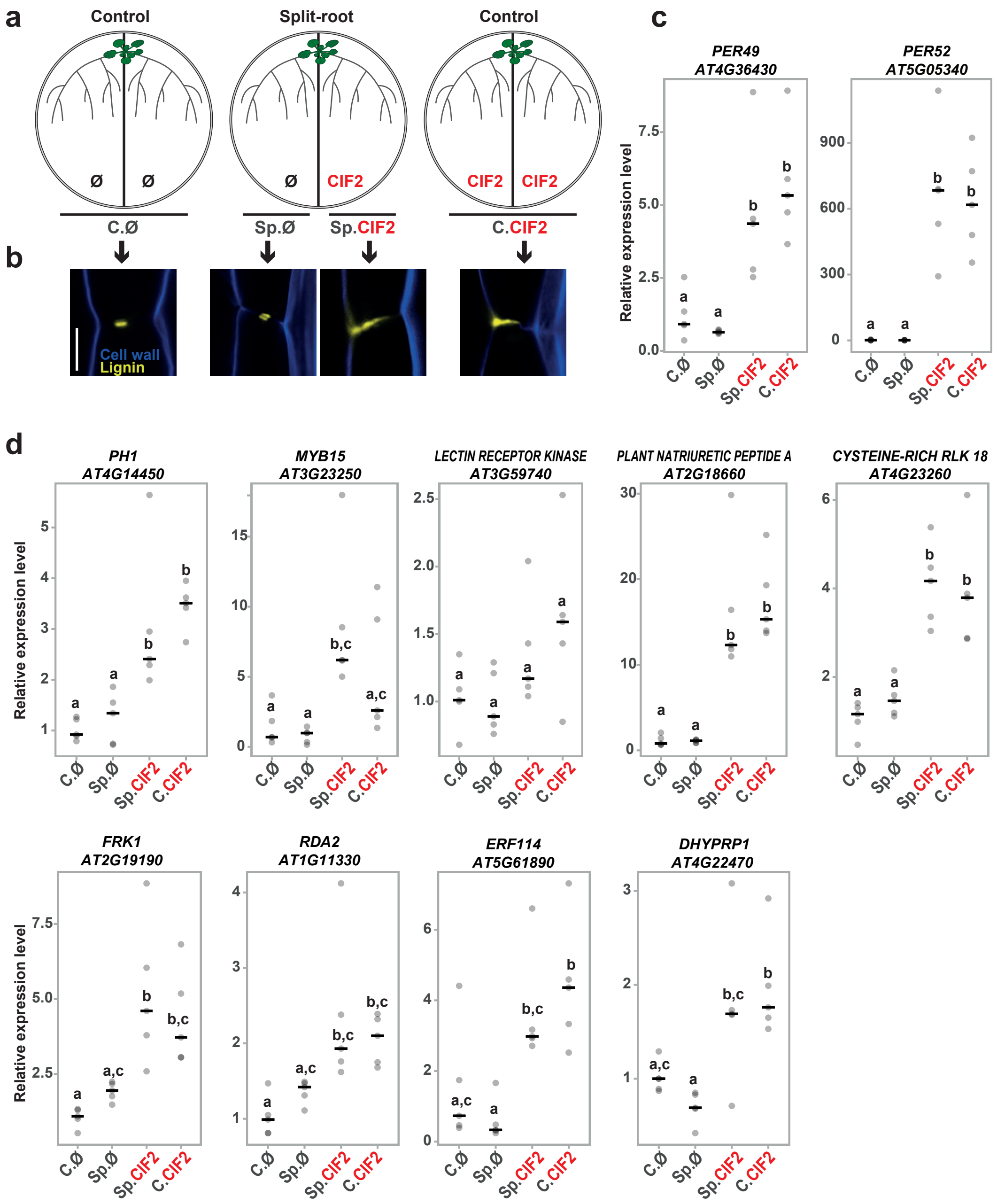

Supplementary Fig. 5. Activation of the Schengen-pathway triggers local lignification and induction of defense-related genes.

a. Scheme of experimental design. Twelve day-old wild-type plants were transferred on round plates containing two compartments. The compartments were filled with control media on both compartments (C.Ø), media supplemented with 100 nM CIF2 on both compartments (C.CIF2) or media only on one side (Sp.Ø) and media supplemented with 100 nM CIF2 (Sp.CIF2) on the other side for the split condition. Plants were grown for 3 more days on these plates and then the roots exposed to each compartment were harvested. **b**. Median view of endodermal cells stained with Direct Yellow 96 (cell wall, blue) and Basic Fuchsin (lignin, yellow). Cells were imaged at 15 cells after the onset of elongation. Scale bar = 10 µm. Representative pictures are shown. Similar results were observed in at least 5 plants. **c**. Gene expression of *Peroxidase 49* and *52* (*PER49* and *52*) determined by qPCR on each compartment described in a. **d**. Gene expression of genes related to defense (*PH1*, *MYB15*, *LECTIN RECEPTOR KINASE*, *PLANT NATRIURETIC PEPTIDE A*, *CYSTEINE-RICH RLK 18*, *FRK1*, *RDA2*, *ERF114* and *DHYPRP1*) determined by qPCR. Different letters in panels a et b indicate significant differences between treatments determined by an ANOVA and Tukey's test as post hoc analyses ($p < 0.05$, $n = 6$ biological replicates). Horizontal black lines indicate median values.

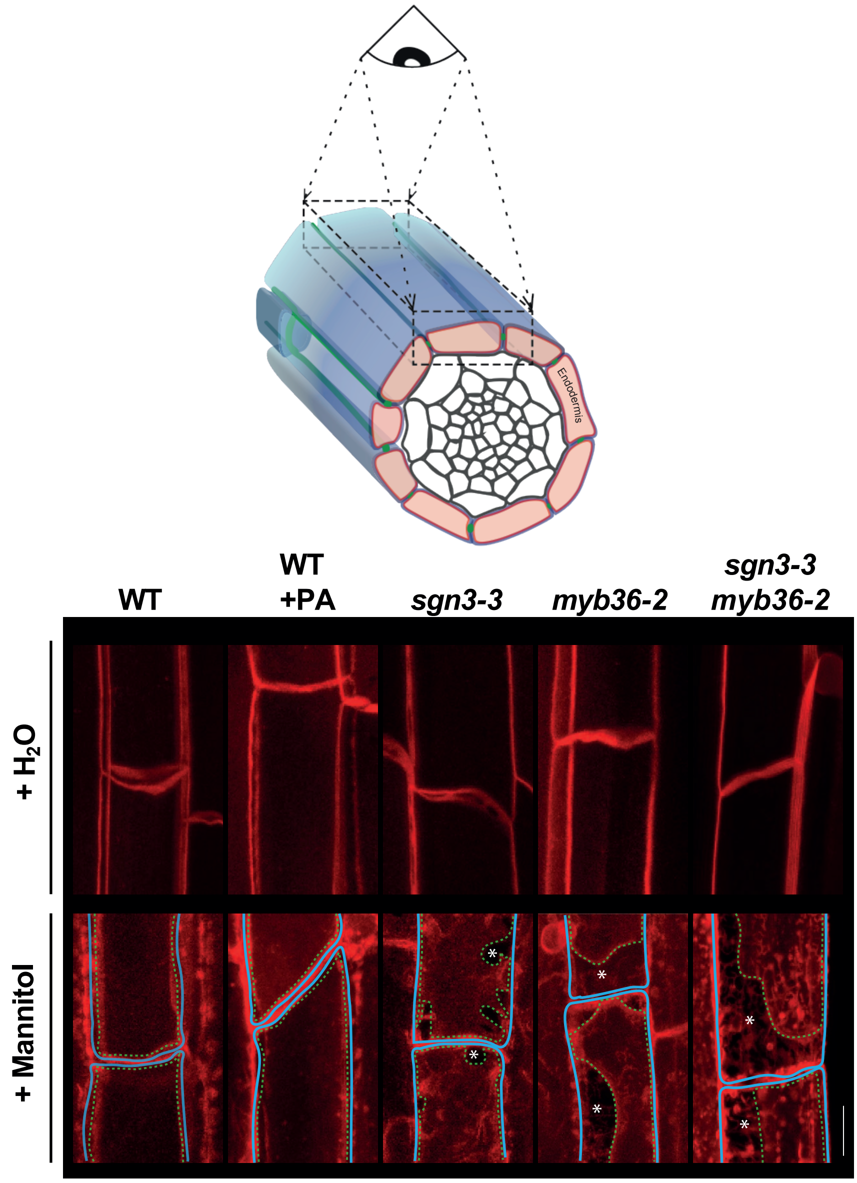

Supplementary Fig. 6. Plasma membrane attachment to the cell wall.
Maximum projection of the top endodermal cells as shown in the schematic view. The observations were done in lines expressing the plasma membrane marker line pELTP::SYP122mCitrine before plasmolysis (+H_2O) and after plasmolysis (+Mannitol) at 15 cells after the onset of elongation. The dashed line represents the contours of the cells. Asterisks show the plasmolysis generated space where no attachment is observed. Scale bar = 5 μm. Representative pictures are shown. The experiment was repeated three times independently with similar results.

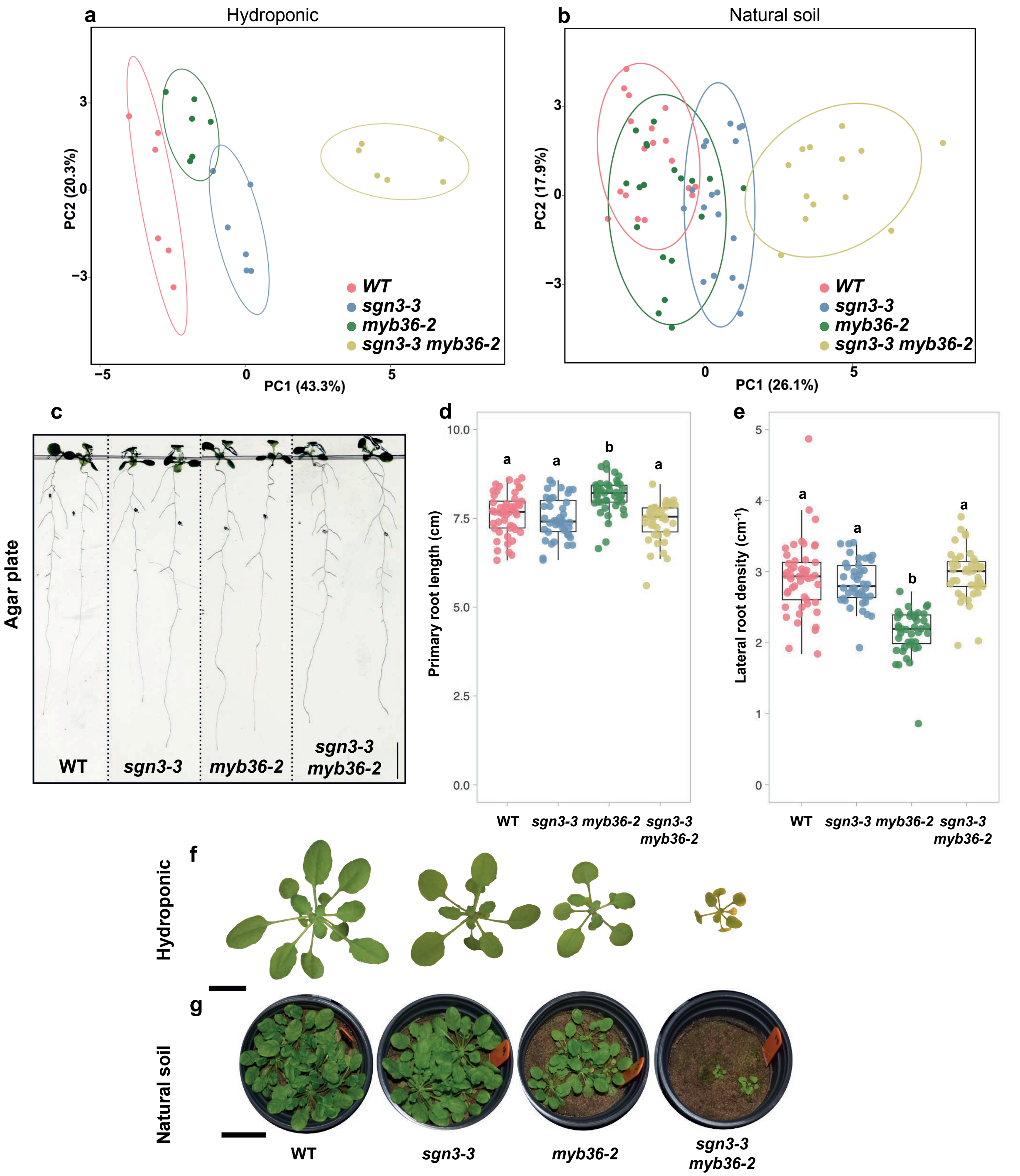

Supplementary Fig. 7. Absence of endodermal apoplastic barrier triggers major ionomic changes in different growth conditions.

a, **b** Principal component analysis (PCA) based on the concentration of 20 elements in shoots of WT, *sgn3-3*, *myb36.3* and *sgn3-3 myb36-2* plants grown in (**a**) hydroponics (short day, n=6) and (**b**) natural soil (short day, n = 18 for WT, n = 18 for *sgn3-3*, n = 18 for *myb36-2* and n = 13 for *sgn3-3 myb36-2*). Ellipses show confidence level at a rate of 90%. **c** Pictures of 2-week-old wild-type (WT), *sgn3-3*, *myb36-2* and *sgn3-3 myb36-2* plants grown in agar plates. **d**, **e** Boxplots showing the primary root length (**d**) and lateral roots density (**e**) of 2-week-old WT, *sgn3-3*, *myb36-2* and *sgn3-3 myb36-2* plants grown in agar plates. Letters show significantly different groups determined by an ANOVA and a Tukey's test as post hoc analyses (n = 49 for WT, n = 42 for *sgn3-3,* n = 43 for *myb36-2* and n = 41 for *sgn3-3 myb36-2*), $p < 0.01$). Center lines show the medians; box limits indicate the 25th and 75th percentiles. **f** Pictures of 5-week-old WT, *sgn3-3*, *myb36-2* and *sgn3-3 myb36-2* plants grown in hydroponics. Scale bar = 1 cm. **g** Pictures of 9-week-old WT, *sgn3-3*, *myb36-2* and *sgn3-3 myb36-2* plants grown in natural soil. Scale bar = 3 cm.

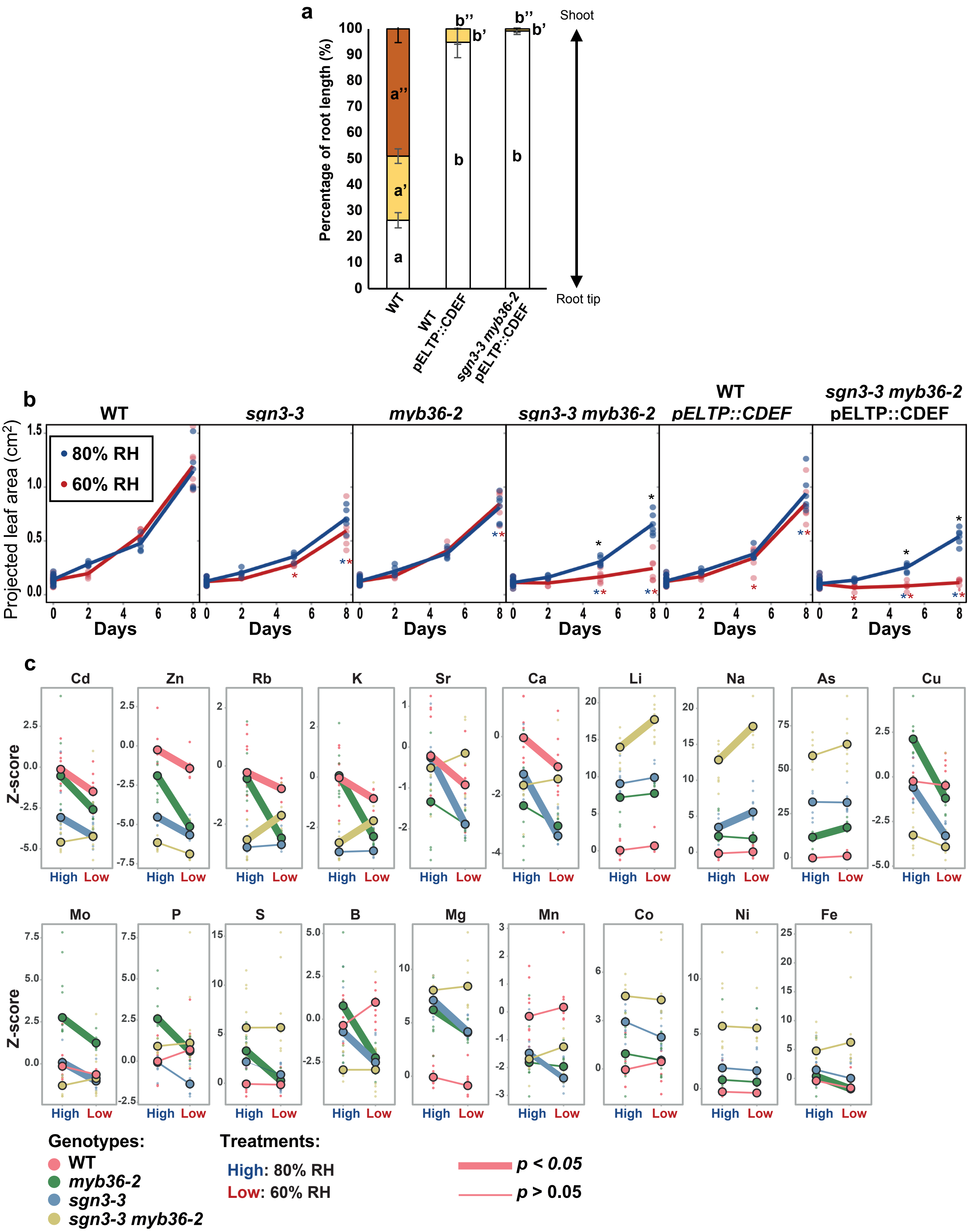
a
Percentage of root length (%)
a
a'
a''
b
b'
b''
Shoot
Root tip
WT
WT pELTP::CDEF
sgn3-3 myb36-2 pELTP::CDEF
b
WT
sgn3-3
myb36-2
sgn3-3 myb36-2
WT pELTP::CDEF
sgn3-3 myb36-2 pELTP::CDEF
Projected leaf area (cm2)
80% RH
60% RH
Days
c
Cd
Zn
Rb
K
Sr
Ca
Li
Na
As
Cu
Mo
P
S
B
Mg
Mn
Co
Ni
Fe
Z-score
High
Low
Genotypes:
WT
myb36-2
sgn3-3
sgn3-3 myb36-2
Treatments:
High: 80% RH
Low: 60% RH
p < 0.05
p > 0.05

Supplementary Fig. 8. Activation of the Schengen-pathway maintains plant growth under fluctuating environment.

a Quantification of suberin staining along the root of 6 days-old plants. The results are expressed in percentage of root length divided in three zones: unsuberised (white), discontinuously suberised (yellow), continuously suberised (orange). n = 7 individual plants, error bars: SD, the centre of the error bars represents the mean. Individual letters show significant differences using a two-sided Mann-Whitney test between the same zones ($p < 0.01$). The experiment was repeated two times independently with similar results. **b** Graphs showing leaf surface area of WT, *sgn3-3*, *myb36-2*, *sgn3-3 myb36-2,* WT-*pELTP::CDEF* and *sgn3-3 myb36-2-pELTP::CDEF* plants germinated in soil with a high humidity (80%) for 7 days and then transferred in an environment with constant (80% RH, blue) or with a lower humidity (60% RH, red). Data were collected at 0, 2, 5 and 8 days after the transfer. Each point is the average leave surface per plant from a single pot (n = 36 pots at 0 day for each genotype, n = 6 pots at 2, 5 and 8 days for each genotype). Each pot contained at least 6 plants for each genotype. The line shows the average value for each measured time points. Black asterisk indicates a significant difference between high and low humidity for a same genotype at one time point. Blue and red asterisk indicate a significant difference in comparison with WT at the same time point respectively for the high and low humidity environment. The significant differences were determined by an ANOVA and a Tukey's test as post hoc analyses (p < 0.01). **c** Graphs showing ions accumulation (Z-score) in shoots of WT, *sgn3-3*, *myb36-2* and *sgn3-3 myb36-2* plants germinated in soil with a high humidity (80%) for 10 days and then transferred in an environment with constant (80% RH, "High" in blue) or with a lower humidity (60% RH, "Low" in red) for 5 more days. Large dots represent the median value for each genotypes and small dots represent individual replicates (n = 9 biological replicates). Thick lines indicate a significant difference using a two-sided *t*-test ($p < 0.05$).

SUPPLEMENTARY REFERENCES

1. Fujita, S. *et al.* SCHENGEN receptor module drives localized ROS production and lignification in plant roots. *The EMBO Journal* **39,** e103894 (2020).
2. Mueller, L. A., Zhang, P. & Rhee, S. Y. AraCyc: a biochemical pathway database for Arabidopsis. *PLANT PHYSIOLOGY* **132,** 453–460 (2003).
3. Liu, J., Osbourn, A. & Ma, P. MYB Transcription Factors as Regulators of Phenylpropanoid Metabolism in Plants. *Molecular Plant* **8,** 689–708 (2015).
4. Ohtani, M. & Demura, T. The quest for transcriptional hubs of lignin biosynthesis: beyond the NAC-MYB-gene regulatory network model. *Current Opinion in Biotechnology* **56,** 82–87 (2018).
5. Raes, J., Rohde, A., Christensen, J. H., Van de Peer, Y. & Boerjan, W. Genome-wide characterization of the lignification toolbox in Arabidopsis. *PLANT PHYSIOLOGY* **133,** 1051–1071 (2003).
6. Huang, J. *et al.* Functional Analysis of the Arabidopsis PAL Gene Family in Plant Growth, Development, and Response to Environmental Stress. *Plant Physiol.* **153,** 1526 (2010).
7. Schilmiller, A. L. *et al.* Mutations in the cinnamate 4-hydroxylase gene impact metabolism, growth and development in Arabidopsis. *The Plant Journal* **60,** 771–782 (2009).
8. Costa, M. A. *et al.* Characterization in vitro and in vivo of the putative multigene 4-coumarate:CoA ligase network in Arabidopsis: syringyl lignin and sinapate/sinapyl alcohol derivative formation. *The International Journal of Plant Biochemistry* **66,** 2072–2091 (2005).
9. Li, Y., Kim, J. I., Pysh, L. & Chapple, C. Four Isoforms of Arabidopsis 4-Coumarate:CoA Ligase Have Overlapping yet Distinct Roles in Phenylpropanoid Metabolism. *PLANT PHYSIOLOGY* **169,** 2409–2421 (2015).
10. Lauvergeat, V. *et al.* Two cinnamoyl-CoA reductase (CCR) genes from Arabidopsis thaliana are differentially expressed during development and in response to infection with pathogenic bacteria. *The International Journal of Plant Biochemistry* **57,** 1187–1195 (2001).
11. Baltas, M. *et al.* Kinetic and inhibition studies of cinnamoyl-CoA reductase 1 from Arabidopsis thaliana. *Plant Physiology et Biochemistry* **43,** 746–753 (2005).
12. Sibout, R. *et al.* CINNAMYL ALCOHOL DEHYDROGENASE-C and -D are the primary genes involved in lignin biosynthesis in the floral stem of Arabidopsis. *THE PLANT CELL ONLINE* **17,** 2059–2076 (2005).
13. Eudes, A. *et al.* Evidence for a role of AtCAD 1 in lignification of elongating stems of Arabidopsis thaliana. *Planta* **225,** 23–39 (2006).
14. Franke, R. *et al.* The Arabidopsis REF8 gene encodes the 3-hydroxylase of phenylpropanoid metabolism. *Plant J.* **30,** 33–45 (2002).
15. Barros, J. *et al.* 4-Coumarate 3-hydroxylase in the lignin biosynthesis pathway is a cytosolic ascorbate peroxidase. *Nature Communications* 1–11 (2019). doi:10.1038/s41467-019-10082-7
16. Do, C.-T. *et al.* Both caffeoyl Coenzyme A 3-O-methyltransferase 1 and caffeic acid O-methyltransferase 1 are involved in redundant functions for lignin, flavonoids and sinapoyl malate biosynthesis in Arabidopsis. *Planta* **226,** 1117–1129 (2007).
17. Hoffmann, L., Maury, S., Martz, F., Geoffroy, P. & Legrand, M. Purification, cloning, and properties of an acyltransferase controlling shikimate and quinate ester intermediates in phenylpropanoid metabolism. *J. Biol. Chem.* **278,** 95–103 (2003).
18. Vanholme, R. *et al.* Caffeoyl shikimate esterase (CSE) is an enzyme in the lignin biosynthetic pathway in Arabidopsis. *Science* **341,** 1103–1106 (2013).

19. Nair, R. B., Bastress, K. L., Ruegger, M. O., Denault, J. W. & Chapple, C. The Arabidopsis thaliana REDUCED EPIDERMAL FLUORESCENCE1 gene encodes an aldehyde dehydrogenase involved in ferulic acid and sinapic acid biosynthesis. *THE PLANT CELL ONLINE* **16,** 544–554 (2004).
20. Kai, K. *et al.* Scopoletin is biosynthesized via ortho-hydroxylation of feruloyl CoA by a 2-oxoglutarate-dependent dioxygenase in Arabidopsis thaliana. *The Plant Journal* **55,** 989–999 (2008).
21. Vanholme, R. *et al.* COSY catalyses trans–cis isomerization and lactonization in the biosynthesis of coumarins. *Nature Plants* 1–12 (2019). doi:10.1038/s41477-019-0510-0
22. Meyer, K., Shirley, A. M., Cusumano, J. C., Bell-Lelong, D. A. & Chapple, C. Lignin monomer composition is determined by the expression of a cytochrome P450-dependent monooxygenase in Arabidopsis. *Proceedings of the National Academy of Sciences* **95,** 6619–6623 (1998).
