# Supplementary material for: Two chemically distinct root lignin barriers control solute and water balance
Source: Nat Commun. 2021 Apr 19;12:2320. doi: 10.1038/s41467-021-22550-0 (PMC8055973; doi:10.1038/s41467-021-22550-0)
Supplement: Supplementary file 3 — Descriptions of Additional Supplementary Files [file 41467_2021_22550_MOESM3_ESM.pdf]

Descriptions of Additional Supplementary Files

Supplementary Data 1
Description: List of the differentially expressed genes in the RNAseq experiment. List of the differentially expressed genes identified in the RNA-seq in root tips of wild-type (WT), sgn3-3, esb1-1, myb36-2, esb1-1 sgn3-3, sgn3-3 myb36-2 plants. Treatment with 100 nM CIF2 was applied as indicated (+CIF2) for WT and sgn3-3 plants. Cluster numbers refer to the clusters defined in Fig. 3a.

Supplementary Data 2
Description: Metabolite profiling in response to the activation of the Schengen-pathway.

Supplementary Data 3
Description: Absence of endodermal apoplastic barrier triggers major ionomic changes. Elemental content in shoot of sgn3-3, myb36-2 and sgn3-3 myb36-2 mutants compared to WT using different growth conditions in agar plates (long day, n=10), in hydroponics (short day, n=6), natural soil (short day, n≥13) and compost at 80 and 60 % relative humidity (RH) (long day, n=9). Element concentration were determined by ICP-MS. Data are presented as mean ± standard deviation (SD). t tests were performed to determine the significant differences to WT for agar plates, hydroponics and natural soil. For compost, t tests were performed to determine the significant differences between 80 and 60 % RH for each genotype. The corresponding p-values are presented.

Supplementary Data 4
Description: List of primers used for qPCR analysis
